# Stable coexistence of equivalent nutrient competitors through niche differentiation in the light spectrum

**DOI:** 10.1002/ecy.2873

**Published:** 2019-09-19

**Authors:** Amanda Burson, Maayke Stomp, Lisette Mekkes, Jef Huisman

**Affiliations:** ^1^ Department of Freshwater and Marine Ecology Institute for Biodiversity and Ecosystem Dynamics University of Amsterdam Amsterdam The Netherlands; ^2^ Marine Biodiversity Group Naturalis Biodiversity Center Leiden The Netherlands; ^3^ Present address: School of Geography University of Nottingham Nottingham United Kingdom

**Keywords:** neutral theory of biodiversity, nitrogen, phosphorus, phytoplankton, resource competition, species coexistence

## Abstract

Niche‐based theories and the neutral theory of biodiversity differ in their predictions of how the species composition of natural communities will respond to changes in nutrient availability. This is an issue of major environmental relevance, as many ecosystems have experienced changes in nitrogen (N) and phosphorus (P) due to anthropogenic manipulation of nutrient loading. To understand how changes in N and P limitation may impact community structure, we conducted laboratory competition experiments using a multispecies phytoplankton community sampled from the North Sea. Results showed that picocyanobacteria (*Cyanobium* sp.) won the competition under N limitation, while picocyanobacteria and nonmotile nanophytoplankton (*Nannochloropsis* sp.) coexisted at equal abundances under P limitation. Additional experiments using isolated monocultures confirmed that *Cyanobium* sp. depleted N to lower levels than *Nannochloropsis* sp., but that both species had nearly identical P requirements, suggesting a potential for neutral coexistence under P‐limited conditions. Pairwise competition experiments with the two isolates seemed to support the consistency of these results, but P limitation resulted in stable species coexistence irrespective of the initial conditions rather than the random drift of species abundances predicted by neutral theory. Comparison of the light absorption spectra indicates that coexistence of the two species was stabilized through differential use of the underwater light spectrum. Our results provide an interesting experimental example of modern coexistence theory, where species were equal competitors in one niche dimension but their competitive traits differed in other niche dimensions, thus enabling stable species coexistence on a single limiting nutrient through niche differentiation in the light spectrum.

## Introduction

Many ecosystems experience major changes in nutrient loads due to anthropogenic eutrophication and the impacts of an expanding human population on land use and climate (Smith 2003, Peñuelas et al. 2012, Glibert et al. 2014). To understand and predict how changes in nutrient loads will affect the species composition of aquatic and terrestrial communities, we may turn to niche‐based theories such as resource competition theory (Tilman 1982, Grover 1997, Brauer et al. 2012). Resource competition theory assumes that species differ in their competitive abilities. The R* value is defined as the lowest possible environmental resource concentration at which a species can still thrive. The species having the lowest R* for a given resource is predicted to be the best competitor for that resource (Tilman 1982). If there are trade‐offs in the competitive abilities of species, such that some species are for example better competitors for nitrogen (N) and others for phosphorus (P), then changes in environmental N:P ratios are expected to lead to predictable changes in species composition.

The neutral theory of biodiversity (Bell 2000, Hubbell 2001) offers an alternative explanation for species diversity that does not adhere to traditional niche differentiation. Instead, neutral theory contends that high diversity is because all species within the same functional group are equivalent in their competitive ability. Random fluctuations in the demographic properties (birth, death, and migration rates) of species are in proportion to their relative abundances in the total community. This results in random ups and downs of population abundances, called ecological drift (Volkov et al. 2003, Hubbell 2005, 2006, Shipley et al. 2006). Hence, neutral theory assumes that species abundances change by chance and not because of differences in competitive abilities (Hubbell 2001, Etienne and Olff 2004).

Although species coexistence in phytoplankton communities has traditionally been interpreted in terms of niche‐based theories (Tilman et al. 1982, Sommer 1985, Stomp et al. 2007, Litchman and Klausmeier 2008, Burson et al. 2018), several recent studies indicate that neutral coexistence might play an important role in phytoplankton communities (Vergnon et al. 2009, Barton et al. 2010, Chust et al. 2013, Segura et al. 2013, Mutshinda et al. 2016, Sakavara et al. 2018). Empirical evidence supporting these ideas is based on field data showing unexplained (random) variation in the relative abundances of species in plankton communities (Chust et al. 2013, Mutshinda et al. 2016), or on clumpy distributions of species traits such as cell size (Vergnon et al. 2009, Segura et al. 2013). In the latter case, the idea is that species within these clumps have similar traits and, hence, their interactions are governed by neutral processes (Segura et al. 2013, Sakavara et al. 2018).

While these results are promising, it is difficult to ascertain from field data whether the species concerned were indeed functionally equivalent. Some relevant environmental or biotic variables may have been overlooked, or species that are similar in some traits (e.g., body size) may be differentiated along other niche dimensions. Unfortunately, controlled experimental studies explicitly identifying and measuring the mechanisms driving species coexistence are relatively rare. The few studies that have rigorously investigated stable coexistence typically utilize invasion experiments, where the long‐term growth of “invading” species into the resident community is tracked to assess mutual invasibility (Huisman et al. 1999, Adler et al. 2006, Narwani et al. 2013, Cothran et al. 2015). Further experimental studies are critical to provide more robust insight into the potential for stable vs. neutral species coexistence, particularly within naturally occurring communities.

In this paper, we investigate whether shifts in N and P loads are likely to result in systematic changes in phytoplankton community composition attributed to niche differentiation or in random changes attributed to neutral competition. For this purpose, we perform controlled competition experiments with a natural phytoplankton community sampled from the North Sea to assess how the species composition responds to changing nutrient loads. The North Sea is a prime example of a marine ecosystem that has witnessed extensive coastal eutrophication from the 1960s to mid‐1980s (Pätsch and Radach 1997, Philippart et al. 2000, Lancelot et al. 2007). During the subsequent de‐eutrophication period, in the past 20–30 yr, riverine P loads into the North Sea have declined more drastically than N loads (Lenhart et al. 2010, Passy et al. 2013, Burson et al. 2016). These changes have created an offshore gradient from P limitation in coastal waters of the North Sea to N limitation in the central North Sea (Burson et al. 2016).

First, we added an inoculum of North Sea phytoplankton to laboratory chemostats to study shifts in species composition in response to different N and P loads. Next, we isolated the two most abundant species from these multi‐species competition experiments and determined their R* values for N and P in monoculture experiments to assess whether they differed in or exhibited similar competitive abilities for these nutrients. Subsequently, we performed pairwise competition experiments in which these two species were inoculated at different initial relative abundances. If the two species would be neutral competitors, then competition experiments starting from different initial conditions should show random drift of the species abundances rather than convergence to the same stable coexistence equilibrium. Conversely, if the two species show niche differentiation, pairwise competition experiments are likely to lead to the same stable coexistence equilibrium, irrespective of the initial relative abundances of the species (although alternative stable states in community composition are also a possibility). Finally, we use the results to evaluate whether the observed species coexistence can be explained by functional equivalence of the species or whether niche differentiation should be invoked.

## Materials and Methods

### North Sea inoculum

Marine phytoplankton was collected from two locations in the North Sea during a research cruise in May 2012 aboard the Dutch research vessel *RV Pelagia* using a sampling rosette equipped with 24 Niskin bottles. Water was sampled from a nearshore station at 7 km from the Dutch coast (53°23′60″ N, 5°9′0″ E) where phytoplankton growth was primarily P limited, and from an offshore station in the central North Sea (56°34′48″ N, 2°10′12″ E) where it was primarily N limited (Burson et al. 2016). At each station, water collected at 7 m depth was passed through an 80‐μm mesh into a 20‐L carboy to remove large zooplankton and debris, and then bubbled for 30 min with N_2_ gas and 30 min with CO_2_ to eliminate smaller grazers while providing inorganic carbon for phytoplankton photosynthesis. The carboys were kept at 4°C until initiation of the chemostat experiments at the University of Amsterdam 2 d after the cruise ended.

### Multispecies community experiments

The phytoplankton communities sampled from the North Sea were grown in laboratory experiments under either N‐limited or P‐limited conditions to investigate which species would become dominant under which nutrient limitation. The experiments were conducted in flat‐walled chemostats (1.7‐L working volume), with full control of light conditions, temperature, pCO_2_ in the gas flow, and nutrient concentrations in the mineral medium (Huisman et al. 1999, Passarge et al. 2006, Ji et al. 2017). Prior to the experiments, the water samples collected from the nearshore and offshore station were mixed in equal proportions, to ensure that the chemostats were inoculated with the same initial community composition. To initiate the experiments, two chemostats were both provided with 0.5 L of the mixed North Sea inoculum and filled up with mineral medium of 35 psu salinity. One of the chemostats received mineral medium with a low N:P ratio of 4:1 (160 μmol/L nitrate, 40 μmol/L phosphate) to induce N limitation, whereas the other chemostat received a high N:P ratio of 60:1 (600 μmol/L nitrate, 10 μmol/L phosphate) to induce P limitation. All other nutrients in the mineral medium were provided at non‐limiting concentrations (Burson et al. 2018).

The front surfaces of the flat chemostat vessels were lit with a constant incident light intensity (*I*_in_) of 40 μmol photons·m^−2^·s^−1^ (PAR range, from 400 to 700 nm), provided by white fluorescent tubes (Philips PL‐L 24W/840/4P, Philips Lighting, Eindhoven, The Netherlands). The chemostat vessels had an optical path length (mixing depth) of 5 cm. Light transmission passing through the chemostats (*I*_out_) was measured daily with a LI‐COR LI‐250 quantum photometer (LI‐COR Biosciences, Lincoln, Nebraska, USA) placed at 10 evenly distributed positions at the back surface of the chemostat vessel.

Inorganic carbon was added as sodium bicarbonate (0.5 mmol/L) in the mineral medium and as CO_2_ mixed into filtered air, which was bubbled through the chemostats at a flow rate of 80 L/h using Brooks instrument pressure flow systems (Hartford, Pennsylvania, USA). The partial pressure of CO_2_ in the air flow was adjusted to maintain a pH of 8.2, which was checked daily with a SCHOTT pH meter (SCHOTT AG, Mainz, Germany). Bubbling of the chemostats further ensured homogeneous mixing of the phytoplankton community, while daily scraping with a magnetic stir bar minimized wall growth. Temperature was maintained at 16°C using cooling plates connected to a thermocryostat, and dilution rates of the chemostats were set at 0.15 per d. Samples for phytoplankton counts were taken three times per week. The multispecies experiments continued until the total biovolume and species composition of the phytoplankton community remained stable for at least 5 d.

### Monoculture and competition experiments

We isolated the two species that became most dominant in the multispecies experiments with the North Sea inoculum using a serial dilution method in 96 well plates, diluting the cell abundances until only one cell per well was deposited. The rRNA gene of the isolated species was amplified for taxonomic identification, using PCR reactions of extracted genomic DNA with 16S rDNA primers for marine cyanobacteria (Nübel et al. 1997) and 18S rDNA primers for marine picoeukaryotes (Moon‐van der Staay et al. 2000) (Appendix S1: Table S1). The PCR products were purified and subsequently sequenced by long run Quick Shot sequencing on an Applied Biosystems 3730XL sequencer (Baseclear, Leiden, The Netherlands). BLAST (Altschul et al. 1990) was used to link the obtained sequences to species names.

Monoculture and competition experiments with the isolated species were conducted in N‐limited and in P‐limited chemostats using the same experimental conditions as described above. R* values (sensu Tilman 1982) for N and P were estimated as the steady‐state concentrations of dissolved inorganic nitrogen (DIN) in the N‐limited monocultures and of dissolved inorganic phosphorus (DIP) in the P‐limited monocultures, respectively. Light absorption spectra of the monocultures were measured at a 0.4‐nm resolution using an AMINCO DW‐2000 double‐beam spectrophotometer (Olis, Bogart, Georgia, USA).

To test whether the initial relative abundances of the species affected the final outcome of competition, the two species were inoculated in the competition experiments at an initial biovolume ratio of 50:1 and at an initial biovolume ratio of 1:50.

In total, this set‐up resulted in two multispecies experiments (one N‐limited and the other P‐limited), four monoculture experiments (two nutrient limitations × two species) and four pairwise competition experiments (two nutrient limitations × two initial conditions). The experiments were not replicated, and one might argue that this is a weakness in our experimental design. However, tailor‐made chemostats and associated laboratory infrastructure are costly, running chemostat experiments is labor intensive, and phytoplankton competition experiments may take several months. This limits opportunities for replication. This is offset by the high degree of experimental precision and control of chemostats, as exemplified by earlier experiments in similar chemostat systems (e.g., Huisman et al. 1999, Passarge et al. 2006, Verspagen et al. 2014). For example, competition experiments with mixtures of five cyanobacterial strains showed almost identical dynamics in triplicate chemostats for >150 d, demonstrating a high degree of reproducibility (Sandrini et al. 2016).

### Phytoplankton and nutrient analysis

In the multispecies experiments, small phytoplankton cells (diameter < 3 μm) were counted using an Accuri C6 flow cytometer (BD Biosciences, San Jose, California, USA) equipped with a blue laser (488 nm) and red laser (640 nm). For this purpose, phytoplankton samples (4.5 mL) were preserved with 0.5 mL formaldehyde (18% v/v)‐hexamine (10% w/v) solution in 5‐mL cryogenic vials. These samples were placed in 4°C for 30 min, flash frozen in liquid nitrogen, and stored at −80°C until flow cytometry analysis. Larger phytoplankton cells (>3 μm) were counted from samples (14 mL) preserved with 1 mL Lugol's iodine and stored in the dark at room temperature until analysis via an inverted microscope (DM IRB, Leica Microsystems, Wetzlar, Germany) using 1‐mL gridded Sedgewick Rafter counting chambers. We counted the entire chamber or 200 cells per species depending on cell concentrations. Biovolumes of the phytoplankton were calculated from cellular dimensions and geometry according to Hillebrand et al. (1999).

In the monoculture and pairwise competition experiments, phytoplankton abundances were quantified as biovolume and as cell numbers using a CASY TTC cell counter (OLS OMNI Life Science, Bremen, Germany), which distinguished between the two species based on their cell size.

Nutrient samples (15 mL) were gently filtered over a 0.22‐μm polycarbonate filter into 20‐mL polyethylene vials and stored in the dark at −20°C until analysis. Nutrients were analyzed using standard colorimetric methods for nitrate and nitrite (Grasshoff et al. 1999), ammonium (Helder and de Vries 1979), and dissolved inorganic phosphorus (DIP; Murphy and Riley 1962). Dissolved inorganic nitrogen (DIN) was defined as the sum of nitrate, nitrite, and ammonium. Cellular nutrients contents were estimated from the total amount of nutrients consumed by the organisms (i.e., the difference between the dissolved inorganic nutrient concentration in the mineral medium supplied to the chemostat and in the chemostat vessel itself) and the measured cell numbers.

## Results

### Multispecies community experiments

**Figure 1:**
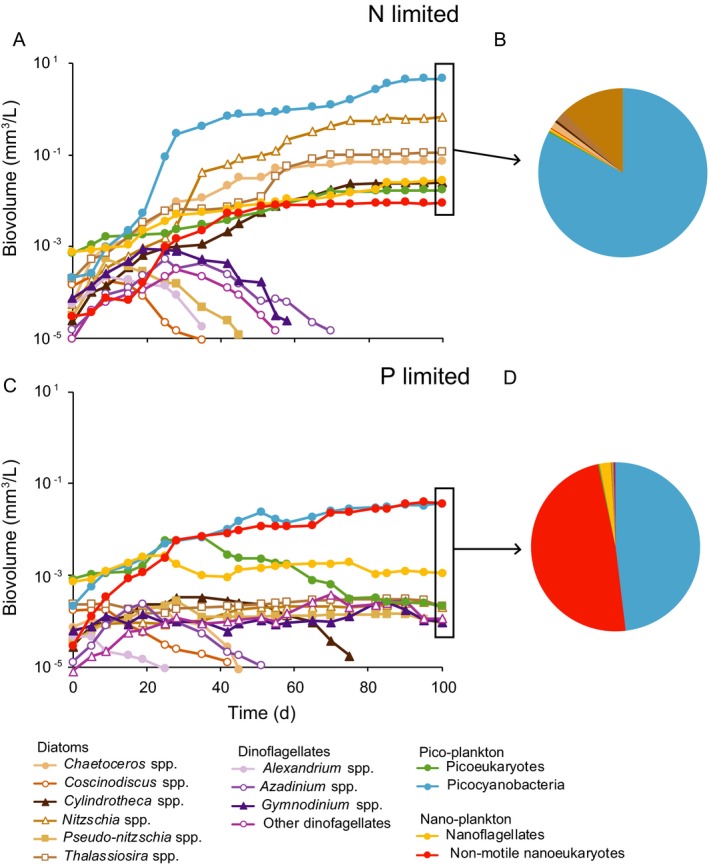
Multispecies community experiments with a phytoplankton mixture sampled from the North Sea. (A) Population dynamics and (B) final community composition in the N‐limited chemostat, where mineral medium was supplied with a molar N:P ratio of 4:1. (C) Population dynamics and (D) final community composition in the P‐limited chemostat, where mineral medium was supplied with a molar N:P ratio of 60:1.

The phytoplankton mixture sampled from the North Sea and used as inoculum for the multispecies experiments consisted of a species‐rich community of nanoflagellates (31% of total biovolume), picoeukaryotes (30%), and diatoms (21%), with smaller contributions by dinoflagellates, nonmotile nanoeukaryotes, and picocyanobacteria. During the first few weeks, almost all species increased in biovolume, indicating that the experimental design initially provided suitable growth conditions for most species in this multispecies community (Fig. 1). Subsequently, after 20–60 d of growth in the N‐limited chemostat, several species were gradually displaced and in the end the N‐limited chemostat was dominated by picocyanobacteria (83%) with a smaller contribution by a diverse group of diatoms (16%; Fig. 1A, B). The P‐limited chemostat also showed competitive exclusion of several species and converged to stable co‐dominance of picocyanobacteria (48%) and nonmotile nanoeukaryotes (48%; Fig. 1C, D).

By means of serial dilution, we isolated the dominant picocyanobacterium from the N‐limited chemostat and the nonmotile nanoeukaryote from the P‐limited chemostat. The 16S rRNA gene sequence of the picocyanobacterium belonged to the *Synechococcus*/*Cyanobium* group, giving a 100% match with 16 strains of *Cyanobium* sp. and two *Synechococcus* sp. strains. The 18S rRNA gene sequence of the nanoeukaryote resulted in a 100% match with the eustigmatophyte *Nannochloropsis* sp. The sequences have been deposited in GenBank and are available under accession numbers KP762160 and KP762161 for *Nannochloropsis* sp. and *Cyanobium* sp., respectively.

### Monoculture experiments

**Figure 2:**
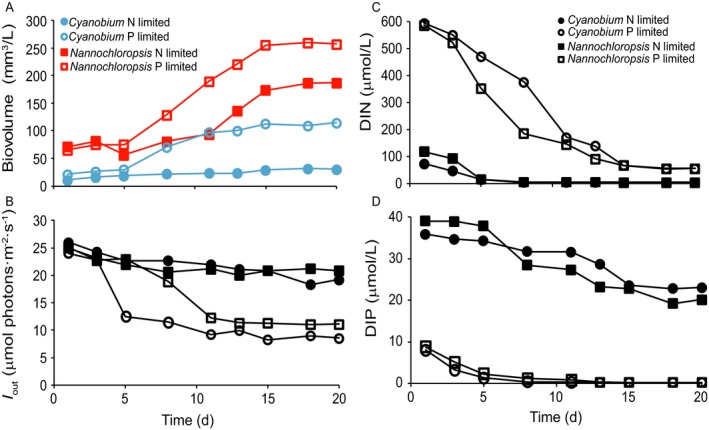
Monoculture experiments of *Nannochloropsis* and *Cyanobium* under N‐limited and P‐limited conditions. (A) Population dynamics of the species, (B) light transmission (*I*_out_) through the monocultures, (C) dissolved inorganic nitrogen (DIN) concentrations, and (D) dissolved inorganic phosphorus (DIP) concentrations in each of the four monoculture experiments.

In monoculture experiments with the isolated *Nannochloropsis* sp. and *Cyanobium* sp., the phytoplankton populations increased (Fig. 2A) while light transmission through the cultures, DIN concentrations and DIP concentrations decreased until a steady state was reached after ~15 d (Fig. 2B–D). *Nannochloropsis* had lower cellular N and P contents than *Cyanobium* (Table 1), and reached a much higher total biovolume than *Cyanobium* under both N‐limited and P‐limited conditions (Fig. 2A). For both species, the steady‐state biovolume was higher in the P‐limited than in the N‐limited monoculture. Accordingly, light transmission through the chemostats was reduced to *I*_out_ ≈ 20 μmol photons·m^−2^·s^−1^ in the N‐limited monocultures but to lower levels of *I*_out_ ≈ 10 μmol photons·m^−2^·s^−1^ in the P‐limited monocultures (Fig. 2B).

**Table 1:** Nutrient requirements of *Cyanobium* and *Nannochloropsis* estimated from the monoculture experiments

Parameter	*Cyanobium*	*Nannochloropsis*
Cell volume (μm^3^)	0.47 ± 0.07	8.55 ± 1.37
Cellular N content		
Per biovolume (pmol/μm^3^)	5.15 ± 0.00	0.85 ± 0.04
Per cell (pmol/cell)	2.17 ± 0.17	6.12 ± 0.30
Cellular P content		
Per biovolume (pmol/μm^3^)	0.087 ± 0.002	0.038 ± 0.000
Per cell (pmol/cell)	0.049 ± 0.007	0.377 ± 0.008
R* value		
For N (μmol/L)	4.85 ± 0.14	5.43 ± 0.03
For P (μmol/L)	0.23 ± 0.02	0.22 ± 0.02

Notes

All values are based on the mean ± SD of the last five time points of the steady‐state monocultures. Cellular N content and R* for N were determined in N‐limited monocultures and cellular P content and R* for P were determined in P‐limited monocultures.

In line with expectation, DIN concentrations were depleted to lower levels in the N‐limited than in the P‐limited monocultures (Fig. 2C). In the N‐limited monocultures, *Cyanobium* depleted DIN to a lower steady‐state concentration (4.85 μmol/L) than *Nannochloropsis* (5.43 μmol/L) (Table 1). Hence, *Cyanobium* had a lower R* value for nitrogen, and is predicted to be a better competitor for nitrogen.

Conversely, DIP concentrations were depleted to lower levels in the P‐limited than in the N‐limited monocultures (Fig. 2D). *Cyanobium* and *Nannochloropsis* depleted DIP to similar concentrations of 0.23 and 0.22 μmol/L, respectively (Table 1). Hence, the two species had similar R* values for phosphorus and are predicted to be equal competitors for P.

### Pairwise competition experiments

**Figure 3:**
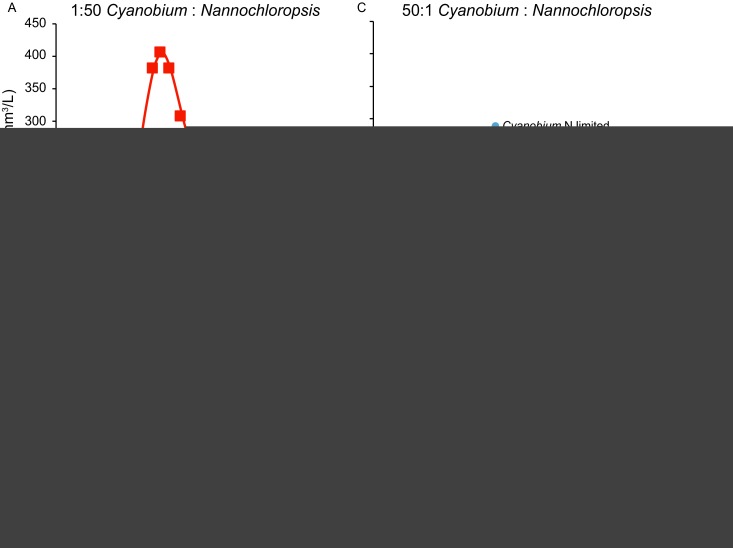
Competition experiments between *Nannochloropsis* and *Cyanobium* under N‐limited conditions. (A, B) Time courses of (A) the competing species and (B) their resources when *Nannochloropsis* was inoculated with a 50× higher initial biovolume than *Cyanobium*. (C, D) Time courses of (C) the competing species and (D) their resources when *Cyanobium* was inoculated with a 50× higher initial biovolume than *Nannochloropsis*.

In the two competition experiments under N‐limited conditions, the time course of competition strongly depended on the initial conditions but the final outcome was the same (Fig. 3). When *Nannochloropsis* was inoculated with a 50× higher initial biovolume than *Cyanobium*,* Nannochloropsis* dominated the experiment for more than 1 month, reaching a very high peak abundance at day 26 (Fig. 3A). A few days after DIN was depleted below 5.4 μmol/L; however, *Nannochloropsis* started to decline and in the end it was competitively excluded by *Cyanobium* (Fig. 3A, B). Conversely, when *Cyanobium* was inoculated with a 50× higher initial biovolume than *Nannochloropsis*,* Cyanobium* maintained its dominance throughout the experiment while *Nannochloropsis* was excluded (Fig. 3C, D).

**Figure 4:**
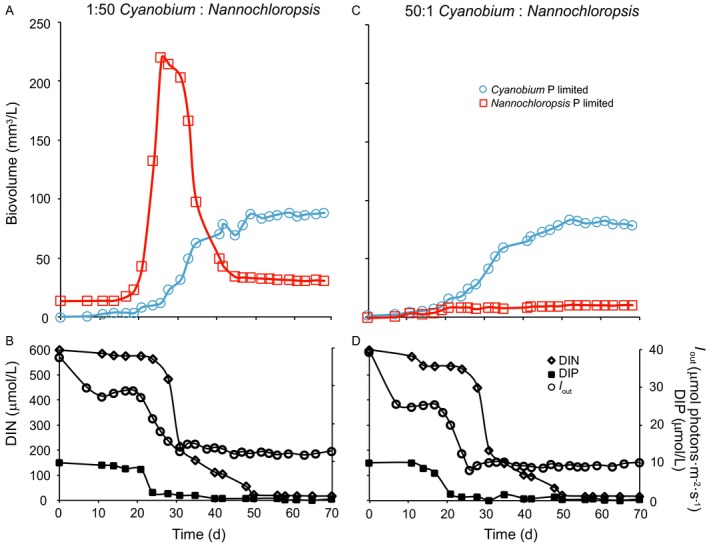
Competition experiments between *Nannochloropsis* and *Cyanobium* under P‐limited conditions. (A, B) Time courses of (A) the competing species and (B) their resources when *Nannochloropsis* was inoculated with a 50× higher initial biovolume than *Cyanobium*. (C, D) Time courses of (C) the competing species and (D) their resources when *Cyanobium* was inoculated with a 50× higher initial biovolume than *Nannochloropsis*.

Under P‐limited conditions, the time course of competition again depended on the initial conditions, but now both species coexisted throughout the experiments (Fig. 4). When *Nannochloropsis* was inoculated with a 50× higher initial biovolume than *Cyanobium*,* Nannochloropsis* again dominated the competition experiment during the first month and reached its peak abundance at day 24 (Fig. 4A). A few days after the DIP concentration was depleted; however, *Nannochloropsis* started to decline while *Cyanobium* increased. Once light transmission through the cultures had been brought down to *I*_out_ ≈ 12 μmol photons·m^−2^·s^−1^ and the DIN concentration had been reduced to ~18 μmol/L, the *Nannochloropsis* and *Cyanobium* populations stabilized and the two species maintained a stable coexistence until the end of the experiment (Fig. 4A, B). Conversely, when *Cyanobium* was inoculated with a 50× higher initial biovolume than *Nannochloropsis*, both species initially increased and then also converged to stable coexistence (Fig. 4C). The *Nannochloropsis* population stabilized from day 22 onward when light transmission through the cultures had been brought down to *I*_out_ ≈ 10 μmol photons·m^−2^·s^−1^, while *Cyanobium* continued to increase for several weeks and reached a stable population from day 50 onward when the DIN concentration had been reduced to ~18 μmol/L (Fig. 4C, D).

## Discussion

### Consistency of results

Our results show dominance of a single species under N‐limited conditions, but stable coexistence of two species under P‐limited conditions. More specifically, under N‐limited conditions, picocyanobacteria became dominant in the multispecies experiments (Fig. 1) and the picocyanobacterium *Cyanobium* competitively displaced the nanoeukaryote *Nannochloropsis* in the pairwise competition experiments (Fig. 3). Under P‐limited conditions, picocyanobacteria and nonmotile nanoeukaryotes co‐dominated in the multispecies experiments (Fig. 1) and *Cyanobium* and *Nannochloropsis* developed a stable coexistence irrespective of their initial relative abundances in the pairwise competition experiments (Fig. 4). These results indicate that picocyanobacteria and nanoeukaryotes were not neutral competitors, but that their population dynamics were governed by interspecific differences in competitive traits.

For each nutrient treatment, we ran one multispecies competition experiment and two pairwise competition experiments starting from different initial species abundances. These three competition experiments are expected to show different transient dynamics, but if all three experiments converge to the same final species composition at steady state, then we can conclude that the final outcome of the experiments is (1) reproducible and (2) independent of the initial conditions. The data of the three competition experiments under N limitation and of the three competition experiments under P limitation confirm these expectations, demonstrating the consistency of the experimental results.

### Competitive exclusion under N limitation

Competitive replacement of *Nannochloropsis* by the picocyanobacterium *Cyanobium* under N‐limited conditions is in line with the lower R* value for N measured in the monoculture of *Cyanobium*. Interestingly, when *Nannochloropsis* was inoculated at a higher relative abundance than *Cyanobium*, it developed an unexpectedly high abundance prior to its competitive replacement by *Cyanobium* (Fig. 3A). *Nannochloropsis* is known to accumulate high concentrations of lipids including polyunsaturated fatty acids under nutrient‐limited conditions, which makes it a high‐quality food source for zooplankton and fish larvae (Krienitz and Wirth 2006) and a species of key interest in biotechnological applications (Pal et al. 2011, Benvenuti et al. 2015). Its high lipid storage explains why *Nannochloropsis* had a low cellular N content per unit biovolume in comparison to *Cyanobium* (Table 1), and hence, why *Nannochloropsis* can produce a much higher biomass per unit N than *Cyanobium*. These results demonstrate that the capacity of a species to produce high biomass does not necessarily provide a competitive advantage, because in the end *Nannochloropsis* lost the competition for N from *Cyanobium*.

### Stable coexistence under P limitation

Resource competition theory predicts that, if species compete for a single resource, the species with lowest R* value for that resource should be the superior competitor (Armstrong and McGehee 1980, Tilman 1982). Our monoculture experiments showed that *Cyanobium* and *Nannochloropsis* had very similar R* values for P. Hence, these two species are predicted to be (nearly) equivalent competitors for P. In this case, neutral theory might apply, according to which the relative abundances of two equivalent competitors would drift more or less randomly rather than converge to competitive exclusion (Hubbell 2001). A classic example is provided by competition experiments of Hansen and Hubbell (1980), where two bacteria coexisted in a neutral fashion when they had similar R* values for the limiting resource tryptophan.

Contrary to these expectations, our results show smooth convergence toward species coexistence instead of randomly drifting species abundances. Moreover, even though the P‐limited competition experiments started from very different initial species abundances, they ultimately led to the same equilibrium outcome. These results clearly point at stable species coexistence with a consistent final outcome irrespective of the initial conditions, rather than the random ecological drift associated with neutral species coexistence.

### Mechanisms of coexistence

Which underlying mechanism allows for stable coexistence of the two species? We note that DIN was reduced to relatively low concentrations of ~18 μmol/L in the P‐limited competition experiments. Although this concentration remained above the low DIN concentrations in the N‐limited competition experiments, it is sufficiently low to affect the growth rates of the species. Hence, co‐limitation by N and P might have affected the species interactions in the P‐limited competition experiments. The standard resource competition model for two essential nutrients (Tilman 1982) predicts stable coexistence of two species if one of these species is a better competitor for P and the other a better competitor for N. The N‐limited competition experiments showed that *Cyanobium* was the better competitor for N. This reasoning therefore assumes that the two species are not identical competitors for P but that the small difference in the R* for P is real, such that *Nannochloropsis* is the better competitor for P. If so, resource competition theory predicts that the DIN concentration at the coexistence equilibrium should have been depleted to the R* value for N of *Nannochloropsis* (~5.4 μmol/L). This was not the case in our experiments, however, as the DIN concentration remained much higher. Hence, co‐limitation by P and N is unlikely to explain species coexistence in the P‐limited competition experiments.

**Figure 5:**
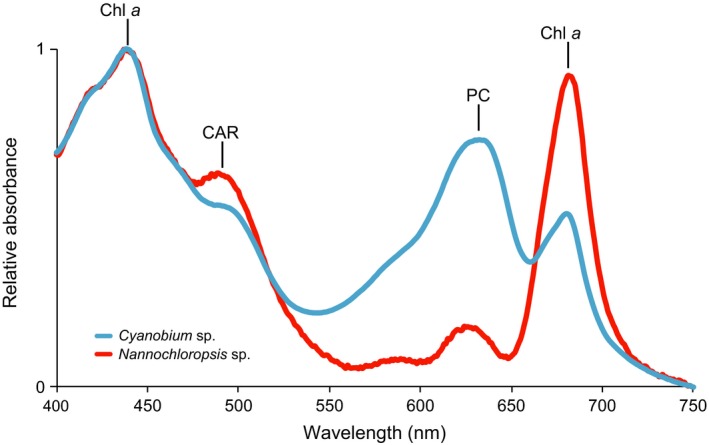
Light absorption spectra of *Nannochloropsis* (red) and *Cyanobium* (blue). Both species contain chlorophyll *a* (Chl *a*), absorbing at 440 and 680 nm. In addition, *Nannochloropsis* contains high contents of carotenoids (CAR) absorbing at 400–520 nm, whereas *Cyanobium* contains carotenoids and the phycobili‐protein phycocyanin (PC) absorbing at 630 nm. The spectra were obtained under nutrient replete conditions.

Furthermore, light intensity was reduced to low levels of 10–12 μmol photons m^−2^ s^−1^ in the P‐limited chemostats (Fig. 4), and hence light may have become a limiting factor for phytoplankton growth. Theoretical and experimental studies have shown that phytoplankton species can exhibit stable coexistence by utilizing different wavelengths of the light spectrum (Stomp et al. 2004, 2007). We measured light absorption spectra of the two species to investigate this possibility. The absorption spectra of *Cyanobium* and *Nannochloropsis* show distinct differences at longer wavelengths (Fig. 5). Specifically, *Nannochloropsis* hardly absorbs yellow and orange light (560–650 nm range), thus leaving these wavelengths freely available for other species to exploit. Conversely, cyanobacteria use phycobilisomes containing stacks of accessory pigments, in this case phycocyanin, which is responsible for the large absorption peak of *Cyanobium* in the orange part of the spectrum at 600–650 nm (Stomp et al. 2004, Haverkamp et al. 2009). Additionally, the chlorophyll *a* peak in the red part (660–700 nm) of the light spectrum is much higher for *Nannochloropsis* than for *Cyanobium* (Fig. 5). Also, although the absorption spectra of *Nannochloropsis* and *Cyanobium* overlap in the blue part of the spectrum (400–470 nm range), recent work shows that species with chlorophyll‐based light‐harvesting complexes such as *Nannochloropsis* use absorbed blue light much more efficiently than cyanobacteria with phycobilisomes as light‐harvesting antennae such as *Cyanobium* (Luimstra et al. 2018, 2019). Hence, in total, *Cyanobium* is more effective in the utilization of orange light (600–650 nm), whereas *Nannochloropsis* is more effective in the utilization of blue (400–470 nm) and red light (660–700 nm). This partitioning of the light spectrum allows for niche differentiation, and therefore offers a plausible explanation for the stable coexistence of these two species.

The interplay between competition for nutrients and the underwater light spectrum is mediated by an intricate network of physiological interactions between nutrient acquisition and photosynthesis. On one hand, the production of photosynthetic pigments and enzymes involved in light harvesting, photosynthetic electron transport and carbon fixation requires nutrients as main building blocks. On the other hand, nutrient uptake and assimilation require chemical energy (ATP) and reducing power (NADPH) generated by photosynthesis. It is thus conceivable that the competitive ability for nutrients of a species depends on the prevailing underwater light spectrum. In other words, species are likely to have lower R* values for limiting nutrients when grown in light that they can absorb for photosynthesis than when grown in light for which they lack adequate pigmentation. In our experimental system, for example, *Cyanobium* would be expected to have a lower R* for P than *Nannochloropsis* in orange light, while *Nannochloropsis* would be expected to have a lower R* for P than *Cyanobium* in red light. If this hypothesis is correct, niche differentiation in the light spectrum will have major impact on competition for nutrients, in agreement with our experimental results. Further validation of this hypothesis could be obtained by advancing new theory and experiments in which species with different photosynthetic pigments compete for limiting nutrients under a range of different light colors.

Several studies have pointed out that niche‐based and neutral processes are not mutually exclusive (Leibold and McPeek 2006, Adler et al. 2007). Instead, the debate about niche differentiation vs. neutrality can be reframed in terms of the relative importance of stabilizing mechanisms (niche differences) and fitness equivalence (neutrality; Chesson 2000, Leibold and McPeek 2006, Adler et al. 2007). In theory, weak, stabilizing forces are sufficient to enable stable coexistence of species with nearly equal fitness (Chesson 2000). In the context of our experiments, this implies that niche differentiation in the underwater light spectrum may have added a stabilizing factor to the otherwise equivalent competition for P between the two species. Conversely, spectral niche differentiation was insufficient to overcome the much larger differences in competitive ability for N between the two species. Hence, the potential for spectral niche differentiation to serve as stabilizing factor in competition likely depends on the magnitude of interspecific differences in competitive ability for limiting nutrients.

### Comparison with natural communities

The pairwise competition experiments were consistent with our multispecies experiments with a phytoplankton community sampled from the North Sea. In both sets of experiments, picocyanobacteria became dominant under N‐limited conditions, whereas picocyanobacteria coexisted with nonmotile nanoeukaryotes under P‐limited conditions. Furthermore, in another series of multispecies competition experiments with North Sea phytoplankton sampled during a different year, we also found stable coexistence of several species on a single limiting nutrient (Burson et al. 2018). Although we did not determine their R* values, the three taxa that coexisted in those experiments (a picocyanobacterium, diatom, and green alga) also differed in their photosynthetic pigments, and hence differences in light absorption spectra may have explained their stable coexistence (Burson et al. 2018). The overall consistency of this previous study and the current results further support our hypothesis that niche differentiation in the light spectrum may provide a stabilizing mechanism for the coexistence of similar nutrient competitors.

Yet, the species composition obtained in our laboratory competition experiments deviated strongly from the natural phytoplankton community composition of the North Sea and other coastal waters. These differences in species composition might be dismissed as an experimental artifact owing to the highly artificial environments provided by laboratory experiments. However, the competitive replacement of large diatoms and dinoflagellates by small pico‐ and nanophytoplankton observed in our multispecies competition experiments is in line with the common expectation that small cells have a competitive advantage under nutrient‐limited conditions (Raven 1998, Irigoien et al. 2004, Edwards et al. 2011). Furthermore, one of the key differences between our lab experiments and natural waters is that we eliminated the zooplankton community. Hence, our findings support the common idea that size‐dependent grazing by zooplankton plays an important role in the persistence of large phytoplankton species in natural waters (Steiner 2003, Fuchs and Franks 2010; Branco et al., *in press*).

## Conclusions

Our study illustrates that empirical demonstration of the mechanisms of species coexistence is far from trivial. In particular, our findings support the prediction of modern coexistence theory (Chesson 2000, Adler et al. 2007, Ellner et al. 2019) that species that appear to be neutral competitors in one niche dimension (with similar R* for P) can display stable species coexistence, if their competitive traits are differentiated across other niche dimensions (e.g., the light spectrum). Thus, although the use of neutral theory to explain seemingly random species distributions in multispecies communities is tempting, we argue that in‐depth analysis of species traits is required as unexpected niche‐based forces can still be at play.
