# Supplementary material for: Stable coexistence of equivalent nutrient competitors through niche differentiation in the light spectrum
Source: Ecology. 2019 Sep 19;100(12):e02873. doi: 10.1002/ecy.2873 (PMC6916172; doi:10.1002/ecy.2873)
Supplement: Supplementary file 1 [file ECY-100-na-s001.pdf]

**Supporting Information.** A. Burson, M. Stomp, L. Mekkes and J. Huisman. 2019. Stable coexistence of equivalent nutrient competitors through niche differentiation in the light spectrum. *Ecology*.

## Appendix S1

**Table S1.** Primers used to sequence 16S and 18S rDNA of the two isolated species.

| Sequence 5' → 3' (length in bp) | Target gene | Target organism | Expected product size | Reference |
|---|---|---|---|---|
| ACCTGGTTGAT CCTGCCAG (19)<br><br>TGATCCTTCYG CAGGTTCAC (20) | 18S rDNA | Pico- and nano-eukaryotes | 1527 bp | Moon-Van der Staay et al. (2000) |
| CGGACGGGTGAGT AACGCGTGA (22)<br><br>GACTACTGGGGTAT CTAATCCCATT (25) | 16S rDNA | Cyanobacteria | ~663 bp | Nübel et al. (1997) |
